# Effects of Acetylated Veneer on the Natural Weathering Properties of Adhesive-Free Veneer Overlaid Wood‒Plastic Composites

**DOI:** 10.3390/polym12030513

**Published:** 2020-02-27

**Authors:** Ying-Ying Chao, Ke-Chang Hung, Jin-Wei Xu, Tung-Lin Wu, Jyh-Horng Wu

**Affiliations:** 1 Department of Forestry, National Chung Hsing University, Taichung 402, Taiwan; banlieue13k@gmail.com (Y.-Y.C.); d9833004@mail.nchu.edu.tw (K.-C.H.); ecsgunro@gmail.com (J.-W.X.); 2 College of Technology and Master of Science in Computer Science, University of North America, Fairfax, VI 22033, USA; tonywuwu22@gmail.com; 3 Department of Wood Science and Design, National Pingtung University of Science and Technology, Pingtung 912, Taiwan

**Keywords:** acetylation, hot press molding, natural weathering, veneer overlaid wood‒plastic composites (vWPCs), weatherability

## Abstract

The purpose of this study is to investigate the natural weathering properties of unmodified and acetylated veneer overlaid wood‒plastic composites (vWPCs) manufactured by one-step hot press molding. The results show that the water absorption and thickness swelling of vWPC with acetylated veneer were lower than those of unmodified vWPC. In addition, the surface tensile strength of vWPC increased with increasing weight gain of acetylated veneer, and the flexural properties of vWPC were not significantly different. Furthermore, the results of natural weathering demonstrated that not only the photostability but also the modulus of elasticity (MOE) retention ratio and surface tensile strength of vWPC with acetylated veneer were significantly higher than those of vWPC with unmodified veneer. Thus, better dimensional stability, surface tensile strength, and weathering properties can be achieved when the vWPC is made with acetylated veneer, especially those containing veneers with a higher weight percent gain.

## 1. Introduction

In the past few decades, wood‒plastic composites (WPCs) have been used in various fixtures, such as window framing, fencing, roofing, decking, and siding [1]. The global WPC market has experienced significant growth in North America and Europe [2]. Additionally, WPCs have been increasingly the focus of research interest [3,4]. However, WPCs are composed of synthetic polymers and wood particles (or wood fiber), which are subjected by photodegradation upon exposure to sunlight, especially ultraviolet (UV) light. Therefore, the color fading and strength weakening of WPCs can be caused by weathering and restrict the WPCs to certain outdoor applications.

It has long been shown that lignin is liable to photodegrade among constituents of wood, and this leads to radical-induced depolymerization of lignin, hemicellulose, and cellulose at the wood surface [5,6]. Furthermore, the strength losses of wood after weathering are caused by wood swelling and shrinkage after moisture effects [7]. It is well known that the dimensional stability, hydrophobicity, and weatherability or durability of wood can be improved by acetylation [8,9,10]. On the other hand, according to Altenbach [11], efficient load bearing of conventional polymer composites with homogeneous single-layered structures could be achieved when polymer composites have multilayered structures, thereby making polymer composites more valuable. It has been shown that layered particleboard and fiberboard have higher flexural strength and stiffness than homogeneous counterparts at the same density level. Hsu et al. [4] reported that the specific flexural properties of three-layered bamboo‒plastic composite (BPC_3L_) were higher than those of homogeneous single-layered BPC.

Furthermore, Najafi et al. [12] and Adhikary et al. [13] indicated that recycled plastic is usually suitable for manufacturing WPCs. Therefore, to improve the aesthetic appearance, flexural strength, and weathering properties of WPCs, unmodified and various acetylated veneers were applied to the surface of WPCs to manufacture adhesive-free veneer overlaid wood‒plastic composites (vWPCs) by one-step hot press molding in this study. Consequently, the physicomechanical and weathering properties of vWPCs with unmodified and acetylated veneers were compared to evaluate the effectiveness of acetylation as a means of improving the weatherability of vWPCs for outdoor applications.

## 2. Materials and Methods

### 2.1. Materials

Taiwan red pine (*Pinus taiwanensis* Hayata), a fast-growing wood species, was sampled from the experimental forest of the National Chung Hsing University in Nan-Tou County. Wood particles were prepared by hammer milling and sieving; particles between 16 and 24 mesh were selected and used in this study. Defect-free rotary-cut radiata pine (*P. radiata* D. Don.) veneer sheets with a thickness of 2 mm were purchased from Wan Tsai Industry Co., Ltd. (Chiayi, Taiwan). Recycled high-density polyethylene (rHDPE; MFI: 4.20 g/10 min; density: 940 kg/m^3^) was kindly supplied by Horng Gee Co., Ltd. (Changhua, Taiwan). All plastic pellets were ground in an attrition mill to reduce their particle size to less than 20 mesh before composite processing. The chemicals and solvents used in this experiment were purchased from Sigma-Aldrich Chemical Co. (St. Louis, MO, USA).

### 2.2. Acetylation Treatments

Veneers were acetylated with acetic anhydride (AA) using the vapor phase reaction method [14] at a solid/liquid ratio of 2 g/mL. All reactions were conducted at 140 °C for 2–8 h to obtain acetylated veneers with different modification degrees. At the end of the reaction, the acetylated veneers were washed with distilled water for 24 h to remove the reagent residues and byproducts (i.e., acetic acid). Finally, the acetylated veneers were dried at 105 °C for 12 h, and the weight percent gain (WPG) was calculated as follows: WPG (%) = 100(*M*_1_ − *M*_0_)/*M*_0_, where *M*_0_ and *M*_1_ are the oven-dried weights of veneer before and after acetylation, respectively.

### 2.3. Composite Processing

**Figure 1:**
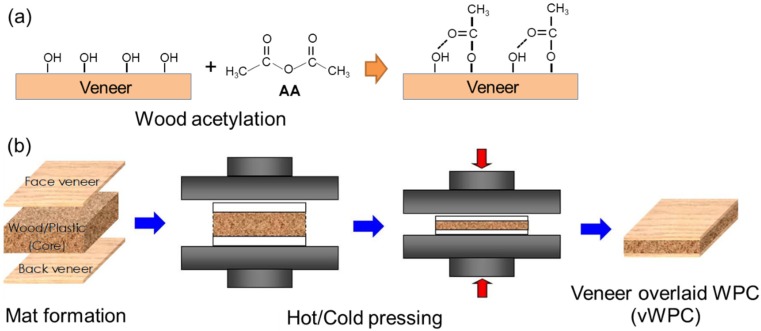
Scheme of (**a**) wood acetylation and (**b**) adhesive-free vWPC processing.

The flat-platen pressing process was applied to the manufacture of adhesive-free vWPCs according to our previous studies [15,16]. The weight ratio of oven-dried wood particles (moisture content less than 3%) to rHDPE powder was 50/50 for the WPC core. The manufacturing process of vWPCs is shown in Figure 1. Two pieces of 2 mm thick veneers were used for the surface layers on both sides of the WPC core mat, and the longitudinal grain directions of the surface veneers were parallel to each other. The expected density of vWPCs was 800 kg/m^3^. The formed sandwich panels (300 mm × 200 mm with 12 mm thickness) were hot pressed at 180 °C and 2.5 MPa for 8 min and then cold pressed until the core temperature of the vWPCs decreased to 40 °C.

### 2.4. Natural Weathering Test

For the natural weathering test, composite species were exposed facing south, inclined at a 45° angle for a period of 1185 days at the campus of National Chung Hsing University (24°07′25.7′′ N, 120°40′30.7′′ E). During exposure periods, the temperature ranged from 7.7 to 36.6 °C, and the average relative humidity and annual precipitation were 77.0% and 6494 mm, respectively. The exposed samples were periodically removed, and their properties were measured regularly.

### 2.5. Determination of vWPC Properties

To determine the properties of the vWPCs, several determinations, including density, water absorption, thickness swelling, flexural properties, and surface tensile strength, were made according to the Chinese National Standard (CNS) 2215. In brief, specimens with dimensions of 230 mm × 50 mm × 12 mm were used to evaluate flexural properties by the three-point static bending test with a loading speed of 10 mm/min and span of 180 mm. The surface tensile strength of the vWPC was determined on samples with dimensions of 50 mm × 50 mm × 12 mm at a tensile speed of 2 mm/min. All samples were conditioned at 20 °C and 65% relative humidity for 2 weeks prior to testing. The retention ratios of modulus of elasticity (MOE) and modulus of rupture (MOR) of the vWPCs after natural weathering were determined as follows: MOE retention ratio (%) = 100(MOE*_t_*/MOE*_0_*); MOR retention ratio (%) = 100(MOR*_t_*/MOR*_0_*), where the measurements with the subscript indices *0* and *t* were for the vWPC data before and after weathering for a time *t*, respectively.

### 2.6. ATR-FTIR Spectral Measurements

Attenuated total reflectance Fourier transform infrared (ATR-FTIR) spectra of all specimens were recorded on a Spectrum 100 FTIR spectrometer (Perkin–Elmer, Buckinghamshire, UK) equipped with a deuterated triglycine sulfate (DTGS) detector and a MIRacle ATR accessory (Pike Technologies, Madison, WI, USA). The spectra were collected by coadding 32 scans at a resolution of 4 cm^−1^ in the range from 650 to 4000 cm^−1^. Three spectra were acquired at room temperature for each composite.

### 2.7. Measurement of Surface Color

The color of the composite surface was measured by a color and color difference meter (CM-3600d, Minolta, Tokyo, Japan) under a D_65_ light source with a test window diameter of 8 mm. The color parameters L*, a*, and b* of all specimens were obtained directly from the colorimeter. Based on the CIE L*a*b* color system, L* is the value on the white/black axis, a* is the value on the red/green axis, b* is the value on the yellow/blue axis, and the ΔE* value is the color difference (ΔE* = [(ΔL*)^2^ + (Δa*)^2^ + (Δb*)^2^]^1/2^).

### 2.8. Analysis of Variance

All results are expressed as the mean ± standard deviation (SD). The significance of differences was calculated by Scheffe’s test or Student’s t-test, and *p* values < 0.05 were considered to be significant.

## 3. Results and Discussion

### 3.1. The Physical and Flexural Properties of vWPCs

The various physical and flexural properties of the vWPC with different WPGs of acetylated veneers are shown in Table 1. The densities of all vWPCs were approximately 785–824 kg/m^3^, and there were no significant differences among them. In addition, after 24 h of immersion in water, the water absorption and thickness swelling decreased with increasing WPG of the veneer. Of these, the vWPC with WPG 16 of acetylated veneers exhibited the lowest water absorption (9.9%) and thickness swelling (1.6%). This phenomenon may be affected by the acetylation of hydroxyl groups of the veneer cell wall with AA, which leads to a decrease in the content of hydrophilic hydroxyl groups and results in more hydrophobic surfaces [17,18]. Another possible explanation for reducing the volume swelling of the acetylated vWPC is that the volume of the veneer cell wall is occupied by the added chemicals (bonded acetyl groups), which results in a decrease in additional swelling of the modified veneer upon exposure to water soaking [19,20].

In addition, Table 1 also shows that there were no significant differences in the modulus of rupture (MOR) and modulus of elasticity (MOE) between unmodified and acetylated vWPCs, even at a WPG of 16%. The values of MOR and MOE for all vWPCs are approximately 46.2–51.9 MPa and 4.1–4.6 GPa, respectively. This result indicated that the flexural properties of the vWPC were not influenced by the acetylation of overlaid veneers. According to the reports of Rowell and Banks [21] and Birkinshaw and Hale [22], acetylation with AA did not noticeably affect the mechanical properties of modified wood. Therefore, the acetylated veneer did not significantly affect the flexural properties of the vWPC. In contrast, the surface tensile strength of the veneer for vWPC increased with increasing WPG of the veneer. The strength increased from the original 490 to 1153 kPa when the WPG of acetylated veneer reached 16%. It is well known that the surface tensile strength of vWPCs depends on the bonding strength between the WPC core and the overlaid veneer. The interfacial adhesion between the veneer and the hydrophobic WPC core can be enhanced by veneer acetylation [18,23,24]. Thus, better stress transfer from the surface veneer to the WPC core through the interface results in high surface tensile strength.

### 3.2. Characteristics of the vWPCs During Natural Weathering

#### 3.2.1. Appearance characteristics of the vWPCs during natural weathering

**Figure 2:**
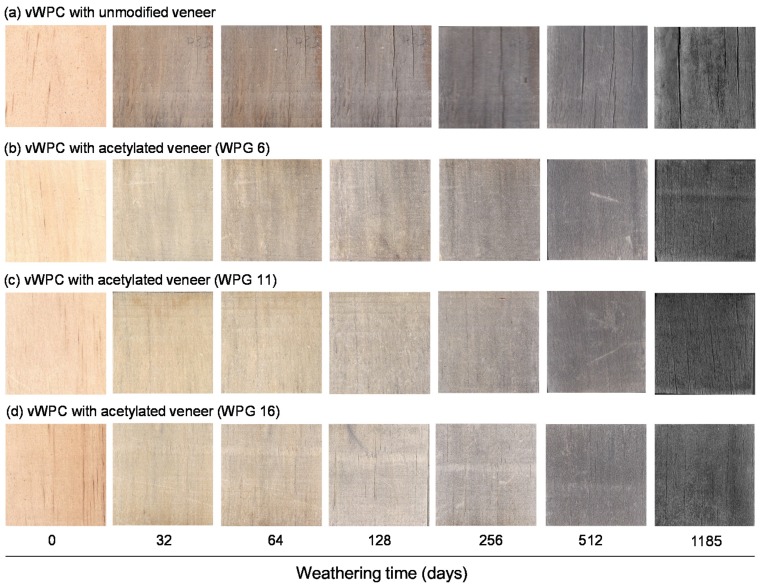
Surface images of vWPCs with unmodified veneers (**a**), WPG 6 (**b**), WPG 11 (**c**), and WPG 16 (**d**) acetylated veneers during 1185 days of natural weathering.

The appearance characteristics of all vWPCs changed significantly during 1185 days of natural weathering. As shown in Figure 2, the surface color of all vWPCs darkens as the natural weathering time increases. In addition, visible cracks developed remarkably at the surface of the veneer for vWPC with unmodified veneer after natural weathering for 32 days. Afterward, the number and size of cracks in unmodified veneer increased with increasing exposure time up to 1185 days of natural weathering. Similar results were also reported by Evans et al. [25]. The explanation given is that the unmodified veneer swelled and shrank after absorbing and desorbing moisture, resulting in cracks at the veneer. In contrast, the vWPCs with acetylated veneers showed almost no crack formation after 1185 days of natural weathering. In other words, the weatherability of the vWPC with acetylated veneers is better than that of the vWPC with unmodified veneers.

#### 3.2.2. Color Changes of the vWPCs During Natural Weathering

**Figure 3:**
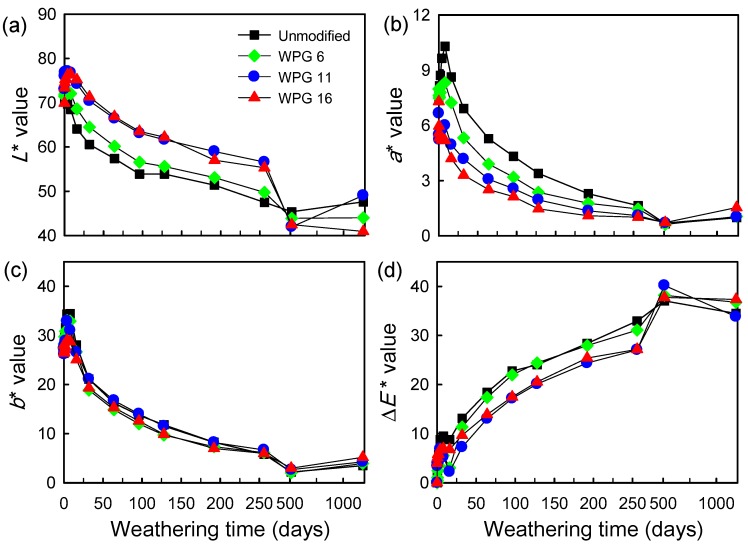
Effect of acetylated veneers on the surface color of vWPCs after natural weathering for 1185 days. (**a**) L*, (**b**) a*, (**c**) b*, and (**d**) ΔE* values. Values are the mean (*n* = 5).

The color variation of vWPCs with unmodified and acetylated (WPG 6, WPG 11 and WPG 16) veneers during 1185 days of natural weathering was evaluated using the CIE L*a*b* color system. As shown in Figure 3, there was no significant difference in the L*, a*, and b* values of all vWPCs before natural weathering. In addition, the L* value of vWPC with unmodified veneer decreased during natural weathering (Figure 3a). This result is different from that of Stark [7], who reported that the lightening of wood floor‒plastic composites occurred during accelerated weathering. However, the L* value of all vWPCs with acetylated veneers increased with increasing exposure time during the first 8 days. Afterward, the value decreased with increasing exposure time. Compared to unmodified vWPC, the L* value of acetylated vWPCs was higher than that of unmodified vWPC after weathering for periods of up to 250 days. In addition, the b* value of all vWPCs showed no significant difference (Figure 3c), but the a* value of unmodified vWPC was higher than that of acetylated vWPC for the same period of time (Figure 3b). After 1185 days of natural weathering, the a* and b* values of all vWPCs had no significant differences. These results revealed that the surface color of unmodified vWPC was darker and redder than that of acetylated vWPC. Meanwhile, Figure 3d shows that all vWPCs exhibited more sensitivity to color change at the initial natural weathering, and the ΔE* values of vWPCs with unmodified, WPG 6, WPG 11, and WPG 16 acetylated veneers were 9.5, 5.6, 6.9, and 6.9, respectively, after weathering for 8 days. Then, the value decreased with increasing exposure time after weathering for periods of up to 32 days. Afterward, the value increased with increasing exposure time until 512 days of natural weathering and then leveling off. The ΔE* values of vWPCs with unmodified, WPG 6, WPG 11, and WPG 16 acetylated veneers were 34.5, 36.8, 33.8, and 37.3, respectively, after natural weathering for 1185 days. This result demonstrated that unmodified vWPC was more susceptible to photooxidation than acetylated vWPCs since acetylated veneers retarded the photodegradation process during the initial period of natural weathering.

It is well known that among the constituents of wood, lignin is most susceptible to photodegradation [26]. Most of the coloring substances generated by photooxidation of lignin come from further reactions between the intermediary phenoxy radicals and oxygen, resulting in the browning process of wood [27]. Therefore, the a* and b* values of vWPC with unmodified veneer increased with increasing exposure time in the first 8 days. However, vWPCs with acetylated veneers retarded the browning process during natural weathering, and similar results were observed on acetylated veneer [8] and esterified wood [28]. Meanwhile, Ohkoshi [29] and Mitsui [30] reported that the acetylated wood subjected by photobleaching upon exposure to UV, resulting in the L* value of all vWPCs with acetylated veneers increased with increasing exposure time in the first 8 days. These results suggest that the acetylation of wood can play a major role in controlling the natural weathering process of wood and wood composites.

#### 3.2.3. Mechanical Properties of the vWPCs During Natural Weathering

The changes in flexural properties and surface tensile strength of various vWPCs during weathering are shown in Table 2 and Table 3, respectively. Table 2 shows that the MOE retention ratios of vWPCs with unmodified and acetylated veneers decreased significantly during natural weathering. Similar results were reported for WPC weathering by Stark [7]. On the other hand, the retained MOE ratio of vWPCs with acetylated veneer (WPG 6, 11 and 16) usually remains at 83.6%–89.5% after natural weathering for 64 days. In contrast, the MOE retention ratio of vWPCs with unmodified veneer was significantly decreased to 48.7% after 64 days of natural weathering. The explanation given is that photodegradation occurs mainly in the lignin on the veneer surface, leading to a cellulose-rich surface. As a result, wood cell walls swell when penetrated by water, facilitating deeper light penetration and providing sites for further degradation, resulting in the deterioration of mechanical properties for veneers [31]. Meanwhile, the unmodified veneer swelled and shrank after absorbing and desorbing moisture. Such cyclic dimensional changes could result in cracks at the veneer (Figure 2), leading to a reduction in the MOE of the veneer and vWPC. However, the dimensional stability and hydrophobicity of wood can be remarkably improved by acetylation [8,9,10], thus resulting in high strength retention for acetylated composites during natural weathering, especially for higher WPG composites. After 1185 days of natural weathering, the MOE retention ratios of the vWPCs with various degrees of acetylation of veneers decreased in the following order: WPG 16 (43.3%), WPG 11 (37.1%), WPG 6 (32.9%), and unmodified (15.8%). Of these, the vWPC with WPG 16 acetylated veneer retained the greatest strength over the weathering period, while the vWPC with unmodified veneer retained the least strength. Similar to the trend observed for the flexural strength, the MOR retention ratios of the vWPCs were WPG 16 (40.2%) > WPG 11 (30.4%) > WPG 6 (21.0%) > unmodified (17.1%) after 1185 days of natural weathering. A similar result was observed on acetylated Scots pine by Even et al. [9].

Furthermore, as shown in Table 3, the surface tensile strength of all vWPCs generally decreased with increasing natural weathering time. Among them, the veneers of all unmodified vWPC were peeled off from the WPC core of vWPC after weathering for 512 days; thus, the unmodified vWPC had not been detected. The explanation for this observation is that the interfacial adhesion between the unmodified veneer and the WPC core is poor, and then the cyclic dimensional changes of veneer during weathering lead to the surface veneer peeling off. However, the interfacial adhesion between the veneer and the WPC core and the dimensional stability of veneers can be enhanced through acetylation. Therefore, the surface tensile strength of vWPC with acetylated veneer remained at 331‒349 kPa after 1185 days of natural weathering. These results demonstrate that the mechanical strength of vWPCs for outdoor application could be improved by veneer acetylation.

#### 3.2.4. ATR-FTIR Analysis of vWPCs During Natural Weathering

**Figure 4:**
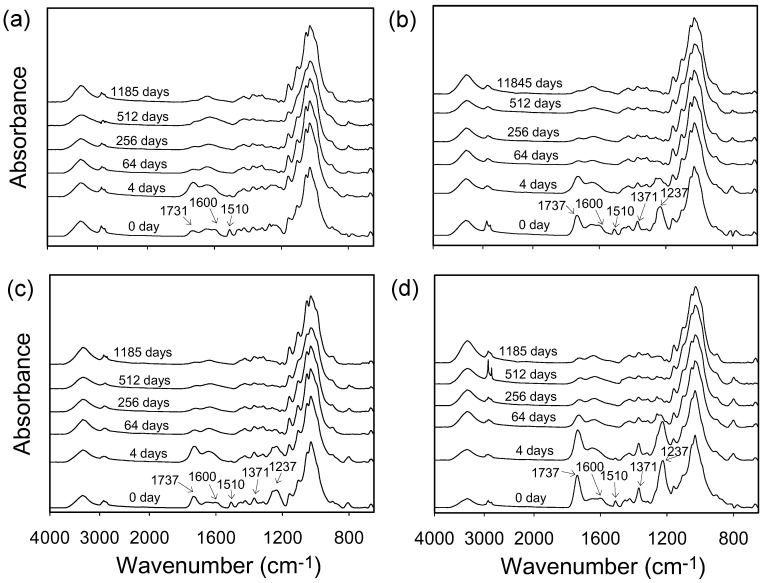
ATR-FTIR spectra of vWPCs with unmodified veneers (**a**), WPG 6 (**b**), WPG 11 (**c**), and WPG 16 (**d**) acetylated veneers during 1185 days of natural weathering.

In this study, ATR-FTIR spectroscopy was used to monitor the specific reactions of vWPCs during natural weathering. As shown in Figure 4, the intensity of absorption bands corresponding to the C=C group in aromatic rings (1510 and 1600 cm^−1^) of lignin decreased with increasing exposure time. After 4 days of natural weathering, the absorption bands of unmodified veneers and acetylated veneers with WPG 6 and 11 almost disappeared. However, the acetylated veneer with WPG 16 retained some of the absorption bands of lignin. Similar results have been reported by Evans et al. [9], who suggested that wood acetylation to lower WPG had no protective effect on lignin and even increased the susceptibility of lignin to degradation during weathering. In addition, the absorption band of the carbonyl (1731 cm^−1^) group of vWPC with unmodified veneer increased significantly in the first 4 days of weathering. Afterward, the absorption band of the carbonyl group decreased or even disappeared as the exposure time increased. Accordingly, an explanation for this phenomenon is that photodegraded products of lignin located on the surface of veneer are leached during weathering, causing the absorption band of carbonyl groups to decrease. Furthermore, the absorption bands at 1737 (–OCOCH_3_, C=O), 1371 (–OCOCH_3_, C–H), and 1237 cm^−1^ (–OCOCH_3_, C–O) also decreased significantly with increasing weathering time for all acetylated vWPCs. This result indicated that the deacetylation or partial hydrolysis of these groups occurred during weathering. Similar results have also been shown in some esterified wood [32]. Accordingly, these results revealed that the effect of acetylation on improving the photostability of vWPCs was not significant.

## 4. Conclusions

Adhesive-free veneer overlaid wood-plastic composites (vWPCs) were successfully prepared by the one-step flat-platen pressing process. The natural weathering properties of vWPCs are greatly influenced by the acetylation of veneers. In this study, vWPCs with acetylated veneers were subjected by photobleaching upon exposure to UV in the first 8 days and retarded the browning process of veneers during natural weathering. However, the ΔE* values of all vWPCs exhibited no significant differences after 1185 days of natural weathering. Of all vWPCs, the vWPCs with a higher degree of acetylation of veneer exhibited higher modulus of elasticity (MOE) and modulus of rupture (MOR) retention ratios after 1185 days of natural weathering, while the vWPCs with unmodified veneer had the lowest retention ratios. In addition, the absorption bands of acetyl groups decreased significantly with increasing weathering time for all acetylated vWPCs. Accordingly, the mechanical property retention of vWPCs can be enhanced through acetylation of veneers, but the effect of acetylation on improving the photostability of vWPCs was not significant. Further research is needed to improve the photostability of vWPCs for outdoor use.

## Figures and Tables

**Table 1:** Effect of acetylated veneers on the physical and flexural properties of vWPCs.

vWPC	Density (MPa)	24 h Soaking		Flexural Properties		Surface Tensile Strength (kPa)
		Water Absorption (%)	Thickness Swelling (%)	MOR (MPa)	MOE (GPa)	
Unmodified	803 ± 18 ^a^	15.6 ± 1.1 ^a^	7.1 ± 0.8 ^a^	50.7 ± 6.1 ^a^	4.6 ± 0.9 ^a^	490 ± 66 ^b^
WPG 6	785 ± 30 ^a^	14.1 ± 0.9 ^ab^	4.7 ± 1.9 ^b^	51.9 ± 3.9 ^a^	4.4 ± 0.5 ^a^	823 ± 83 ^ab^
WPG 11	819 ± 21 ^a^	12.3 ± 1.6 ^bc^	3.2 ± 1.2 ^bc^	47.8 ± 3.2 ^a^	4.1 ± 0.6 ^a^	929 ± 17 ^a^
WPG 16	824 ± 16 ^a^	9.9 ± 1.7 ^c^	1.6 ± 0.2 ^c^	46.2 ± 8.6 ^a^	4.2 ± 0.6 ^a^	1153 ± 103 ^a^

Values are the mean ± SD (*n* = 5 for 24 h soaking and flexural properties; *n* = 3 for surface tensile strength). Different letters indicate significant differences within a column (*p* < 0.05).

**Table 2:** Effect of acetylated veneer on MOE and MOR retention ratio of vWPCs after natural weathering for 1185 days.

vWPC	MOE Retention Ratio (%)					MOR Retention Ratio (%)
	0 days	64 days	256 days	512 days	1185 days	1185 days
Unmodified	100.0 ± 8.4 ^a^	48.7 ± 17.6 ^bB^	38.2 ± 16.6 ^bcA^	30.0 ± 13.7 ^bcA^	15.8 ± 4.7 ^cA^	17.1 ± 4.1 ^B^
WPG 6	100.0 ± 17.2 ^a^	86.2 ± 11.4 ^abA^	49.5 ± 33.5 ^abA^	41.9 ± 29.6 ^bA^	32.9 ± 22.6 ^bA^	21.0 ± 11.4 ^AB^
WPG 11	100.0 ± 11.2 ^a^	83.6 ± 12.5 ^abA^	72.8 ± 30.5 ^abA^	63.4 ± 32.3 ^abA^	37.1 ± 21.8 ^bA^	30.4 ± 11.9 ^AB^
WPG 16	100.0 ± 19.5 ^a^	89.5 ± 7.0 ^aA^	78.5 ± 11.9 ^abA^	67.5 ± 26.1 ^abA^	43.3 ± 17.6 ^bA^	40.2 ± 9.5 ^A^

Values are the mean ± SD (*n* = 5). Different lowercase and capital letters indicate significant differences within a raw and a column (*p* < 0.05), respectively.

**Table 3:** Effect of acetylated veneers on the surface tensile strength of vWPCs after natural weathering for 1185 days.

vWPC	Surface Tensile Strength (kPa)				
	64 days	128 days	256 days	512 days	1185 days
Unmodified	250 ± 31	265 ± 3	295 ± 110	ND	ND
WPG 6	797 ± 34 ^**^	654 ± 237	469 ± 199	367 ± 66	331 ± 30
WPG 11	686 ± 31 ^**^	757 ± 144 ^*^	599 ± 246	515 ± 21	349 ± 23
WPG 16	722 ± 82 ^**^	889 ± 37 ^**^	864 ± 110 ^*^	338 ± 78	342 ± 79

ND: not detected. Values are the mean ± SEM (*n* = 3). ^*^: *p* < 0.05; ^**^: *p* < 0.01 (one-tailed test) compared to the “unmodified” group.
